# Expression of a *Yersinia pseudotuberculosis* Type VI Secretion System Is Responsive to Envelope Stresses through the OmpR Transcriptional Activator

**DOI:** 10.1371/journal.pone.0066615

**Published:** 2013-06-19

**Authors:** Erwan Gueguen, Eric Durand, Xiang Y. Zhang, Quentin d’Amalric, Laure Journet, Eric Cascales

**Affiliations:** Laboratoire d’Ingénierie des Systèmes Macromoléculaires (LISM, UMR 7255), Institut de Microbiologie de la Méditerranée (IMM), Centre National de la Recherche Scientifique (CNRS), Aix-Marseille Université, Marseille, France; Institut Pasteur, France

## Abstract

The Type VI secretion system (T6SS) is a macromolecular complex widespread in Gram-negative bacteria. Although several T6SS are required for virulence towards host models, most are necessary to eliminate competitor bacteria. Other functions, such as resistance to amoeba predation, biofilm formation or adaptation to environmental conditions have also been reported. This multitude of functions is reflected by the large repertoire of regulatory mechanisms shown to control T6SS expression, production or activation. Here, we demonstrate that one T6SS gene cluster encoded within the *Yersinia pseudotuberculosis* genome, T6SS-4, is regulated by OmpR, the response regulator of the two-component system EnvZ-OmpR. We first identified OmpR in a transposon mutagenesis screen. OmpR does not control the expression of the four other *Y. pseudotuberculosis* T6SS gene clusters and of an isolated *vgrG* gene, and responds to osmotic stresses to bind to and activate the T6SS-4 promoter. Finally, we show that T6SS-4 promotes *Y. pseudotuberculosis* survival in high osmolarity conditions and resistance to deoxycholate.

## Introduction

The Type VI secretion system (T6SS) is a macromolecular machine dedicated to the secretion of toxin proteins, widespread in Gram-negative Proteobacteria [1]–[5]. This system is highly versatile as it can target eukaryotic or prokaryotic cells [6]. The *Aeromonas hydrophila* and *Vibrio cholerae* T6SS have been shown to be required for full virulence towards eukaryotic host cells via the transport of protein domains responsible for actin modification [7]–[11]. Several other T6SS, including those of *Pseudomonas aeruginosa*, *Burkholderia thailendensis*, *Serratia marcescens V. cholerae*, *Citrobacter rodentium* and enteroaggregative *Escherichia coli*, are required to eliminate competing bacteria in mixed environments [12]–[17]. The toxins secreted by the *P. aeruginosa* and *S. marcescens* T6SS have been recently identified and characterized: the *Pseudomonas* Tse1, Tse3 and *Serratia* Rab proteins are translocated into the periplasm of the prey cells where they create lesions in the peptidoglycan layer [18]–[20]. Additional T6SS functions have been reported such as role in stress sensing, biofilm formation or adaptation to environmental conditions, although the mechanistic bases for these functions have not been clearly defined [21]–[24].

At a molecular level, the T6SS is assembled via interactions between 13 different components, called Tss proteins [25]–[26]. Several of these proteins share structural homologies with components of the tail of contractile bacteriophages, including the major tail tube protein (Hcp), the cell puncturing device (VgrG), the sheath (TssB-C) and at least one component of the baseplate (TssE) of bacteriophage T4 [27]–[32]. Mechanistically, it has been proposed that this bacteriophage-like complex assemble a tubular structure similar to the bacteriophage tail tube, wrapped by a contractile sheath-like structure. As evidenced for the bacteriophage, contraction of the T6SS sheath will propel the inner tube to the cell exterior. Indeed, recent cryo- and fluorescence microscopy data demonstrated that the TssB-C proteins form dynamic cytoplasmic structures, oscillating between extended and contracted conformations [33] and that T6SS sheath contraction is correlated with prey cell lysis [17]. Several other T6SS proteins are embedded into the inner or outer membranes where they assemble a trans-envelope spanning complex proposed to anchor the bacteriophage-like structure to the envelope [34]–[39].

Although the architecture of the T6SS seems to be conserved, it is however clear that T6SS have been rerouted to be dedicated to functions important to the need of each individual species. This is also clearly reflected by the regulatory mechanisms identified so far [40]–[42]. T6SS activity is regulated at different levels. T6SS gene transcription is dependent on transcriptional activators, two-component systems, histone-like proteins, alternative sigma factors or regulatory RNAs whereas post-translational mechanisms based on the phosphorylation status of a fork-head associated protein modulate T6SS activity [40]–[46]. One interesting observation is that T6SS gene clusters can be found in several copies in bacterial genomes. While many species encode a single locus, 2, 3 or more (up to 6) complete T6SS gene clusters can be scattered on the genome [1], [5]. This is the case of the *Yersinia pseudotuberculosis* genome that encodes four complete and two incomplete machineries [5], [47]. This bacterium constitutes an interesting model to identify the regulatory mechanisms controlling the expression of these T6SS, potential regulatory cross-talks, the specific function of each T6SS and specificity determinants during assembly.

To gain insights into the regulatory mechanisms underlying expression of the *Y. pseudotuberculosis* T6SS gene clusters, we constructed transcriptional *lacZ* and *gfp* reporter fusions to the T6SS-4 gene promoter, a T6SS locus shared by *Y. pseudotuberculosis* and *Y. pestis*. Although the *Y. pestis* CO92 T6SS-4 gene cluster has been shown to promote phagocytosis and to limit intracellular replication in macrophages, it has no role during rat flea infection or in virulence in murine bubonic plague models [48]. As previously reported, the T6SS-4 promoter was more active at low temperature (28°C vs 37°C) [47]–[49]. By using transposon mutagenesis we identified OmpR as a potential regulator. OmpR binds on the T6SS-4 promoter region *in vitro* and does not regulate the other T6SS loci or an isolated *vgrG* gene. OmpR is the response regulator of the EnvZ-OmpR two component systems that is responsive to cell envelope and osmotic stresses [50]–[52]. Interestingly, LptD was also identified in the same screen. LptD is a component of the Lpt machine required for the proper insertion of lipopolysaccharides in the outer leaflet of the outer membrane [53]–[55]. Epistasis experiments showed that LptD acts upstream OmpR suggesting that T6SS-4 expression might be responsive to cell envelope stresses. Indeed, we show that the transcriptional fusion is activated in high osmolarity conditions and in presence of the bile salt deoxycholate, and that T6SS-4 is required for survival after osmotic stress and for increased resistance to deoxycholate.

## Results

### *Yersinia Pseudotuberculosis* T6SS-4 Expression is Thermoregulated

The T6SS-4 gene cluster is found in both *Y. pestis* and *Y. pseudotuberculosis* its expression has been shown to be thermo-dependent in both species [47]–[49]. To gain further insights into the regulatory mechanism underlying T6SS-4 expression, we constructed a *lacZ* fusion to the T6SS-4 promoter at the original locus in strain *Y. pseudotuberculosis* IP31758. To avoid false positive clones during the transposon mutagenesis screen (*e.g.*, transposon insertion into the *lacZ* reporter gene), we introduced a second promoter-*gfp* fusion at the *ara* locus, yielding the IP31758-41 strain. As previously reported in both *Y. pestis* and *Y. pseudotuberculosis*, β-galactosidase activities and GFP fluorescence confirmed that the expression of the T6SS-4 locus is activated at 28°C compared to 37°C (see Fig. 4A).

### Transposon Mutagenesis Identifies *OmpR* and *LptD* as Candidates for T6SS-4 Regulation

**Figure 1:**
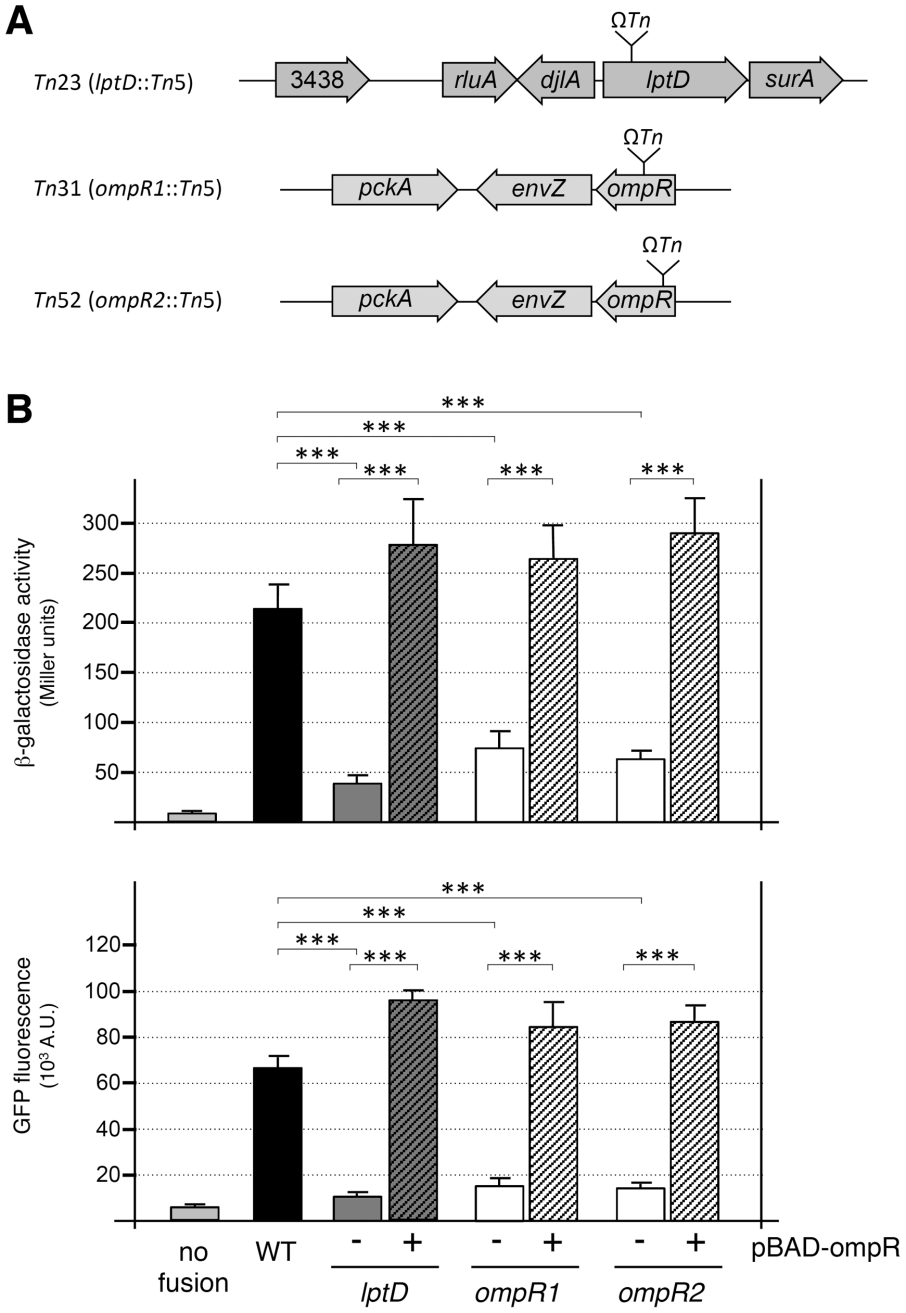
Transposon mutagenesis identified OmpR and LptD as regulators of T6SS-4 expression. (A) Location of the transposon in the three strains (*Tn*23, *Tn*31 and *Tn*52) displaying lower T6SS-4 expression isolated in the random screen. (B) β-galactosidase activities (upper panel, in Miller units) and fluorescence levels (lower panel, in arbitrary units) of *Y. pseudotuberculosis* RL31748-4 (no fusion), *Y.p.* RL31758-41 (WT, carrying the promoter-*lacZ* fusion at the locus and the promoter-*gfp* fusion at the *ara* locus) and of the transposon strains carrying the pBAD24 empty vector (−) or pBAD-ompR (+). *p*-values obtained using paired Student’s *t-*test analyses are indicated (***, *p*≤0.0001).

To identify regulators, we screened mini-*Tn*5 transposon mutants for decreased T6SS-4 expression at 28°C. *Tn*5 transposons insert randomly in the genomes of *Yersinia* species [56]. Approximatively 250,000 transposon mutant strains were screened and 128 clones with a white *lacZ* ^−^ phenotype were isolated on McConkey plates. These 128 transposon mutants were tested for GFP fluorescence levels to eliminate potential insertion into the *lacZ* reporter gene. 31 candidates were retained with both decreased *lacZ* activity and fluorescence levels. The site of mini-*Tn*5 transposon insertion was determined for 3 of these candidates that displayed stronger decreases. One insertion occurred at close proximity to the T6SS-4 locus (YpsIP31758_3437 to YpsIP31758_3420), into the *lptD* gene (YpsIP31758_3441; gene accession YP_001402396.1). Two independent transposon insertions mapped into the *ompR* locus (YpsIP31758_3980; gene accession YP_001402928.1) (Figure 1A).

### OmpR Activates Transcription of the *Y. Pseudotuberculosis* T6SS-4 Locus

OmpR is the response regulator of the two-component EnvZ-OmpR system [57]. To confirm that OmpR is involved in the regulation of the T6SS-4 locus, we cloned the *Y. pseudotuberculosis ompR* gene under the control of the P_BAD_ promoter into pBAD24 [58]. β-galactosidase activities and fluorescence levels of the transposon mutants carrying the empty pBAD24 or the pBAD24-OmpR vectors were measured. Figure 1B shows that the *lptD* mutant displayed a 6-fold decrease of β-galactosidase activity and a 7-fold decrease of fluorescence compared to the wild-type strain whereas the two *ompR* mutant strains (*ompR1* and *ompR2*) displayed a 4-fold decrease of β-galactosidase activity and of fluorescence. The levels of β-galactosidase activity and of fluorescence were complemented by the overproduction of OmpR into the two *ompR* mutant strains, demonstrating that the OmpR protein produced from pBAD24 was fully functional.

To determine whether LptD acts upstream or downstream OmpR in the regulatory cascade we performed epistasis experiments. In the *lptD* strain, overproduction of OmpR led to an increased fluorescence and β-galactosidase activity compared to the *lptD* strain carrying the empty vector, at levels comparable to the WT strain (Figure 1B). These data suggest that OmpR is acting downstream LptD.

**Figure 2:**
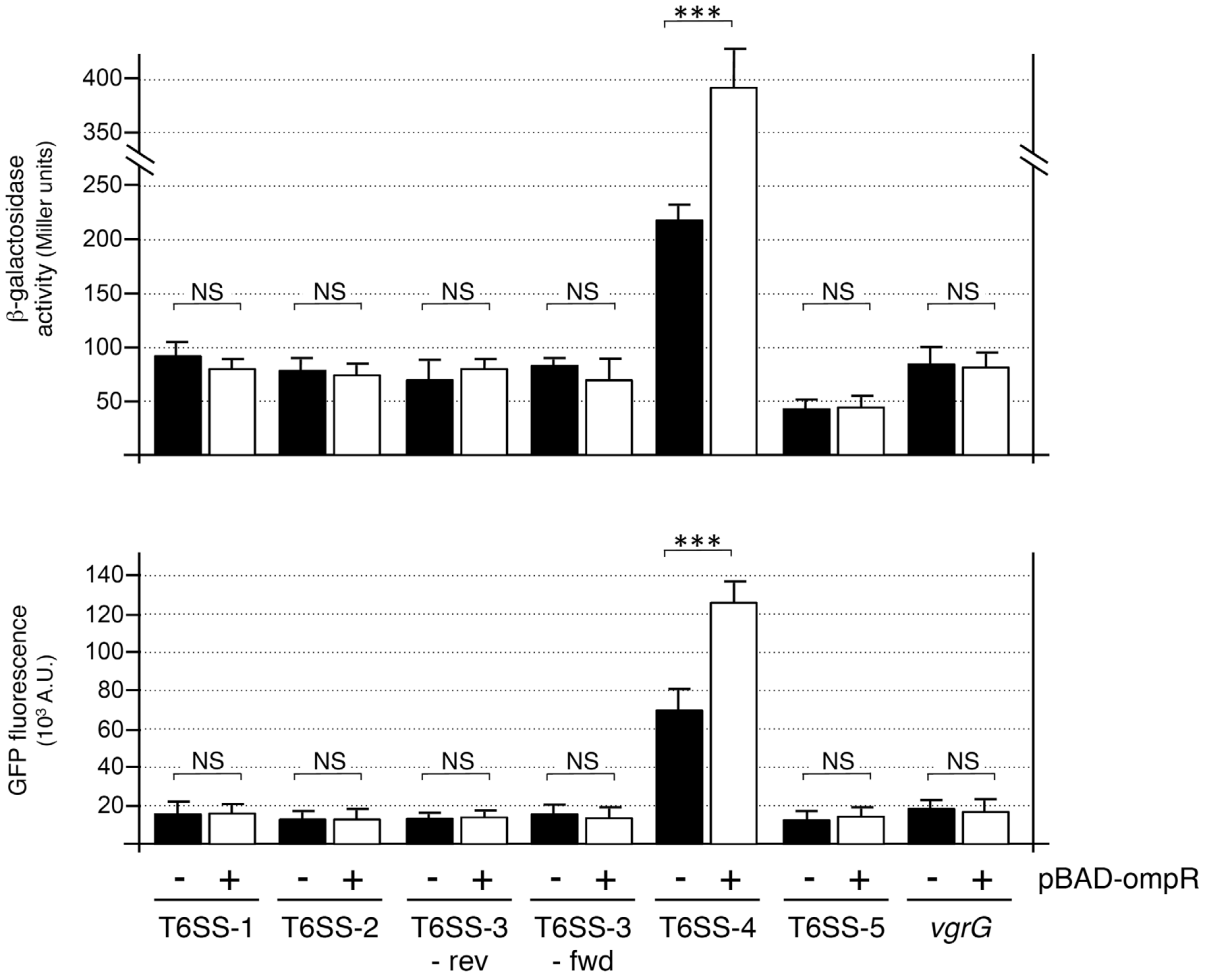
OmpR does not regulate the other *Y. pseudotuberculosis* T6SS loci. β-galactosidase activities (upper panel, in Miller units) and fluorescence levels (lower panel, in arbitrary units) of *Y. pseudotuberculosis* with *lacZ* and *gfp* fusions to T6SS-1, T6SS-2, T6SS-3-rev, T6SS-3-fwd, T6SS-4, T6SS-5 and *vgrG* (IP31758_0696) putative promoters, and carrying the pBAD24 empty vector (−) or pBAD-ompR (+). *p*-values obtained using paired Student’s *t-*test analyses are indicated (NS, non significant [*p*>0.05]; ***, *p*≤0.0001).

We further tested whether OmpR regulates the expression of the four other *Y. pseudotuberculosis* T6SS loci (T6SS-1, YpsIP31758_0312 to _0339; T6SS-2, YpsIP31758_2511 to _2485; T6SS-3, YpsIP31758_1354 to _1379 and T6SS-5, YpsIP31758_0777 to _0805) and of an isolated *vgrG* gene (YpsIP31758_0696). Here again, strains with transcriptional *lacZ* reporter at the locus and promoter-*gfp* reporter at the *araBAD* locus were constructed. The T6SS-3 gene cluster being composed of two divergent operons (YpsIP31758_1361 to _1354 [T6SS-3-rev] and Yps31758_1362 to _1379 [T6SS-3-fwd]), fusions to *lacZ* and *gfp* were introduced in both orientations. Figure 2 shows that the β-galactosidase activity and fluorescence levels of these promoters were very low, suggesting these promoters are weakly expressed in rich medium and low temperature (28°C). Aside T6SS-4, overproduction of the OmpR response regulator in these strains did not affect significantly the activity of the promoters.

### Purified OmpR Binds to the T6SS-4 Promoter Region

**Figure 3:**
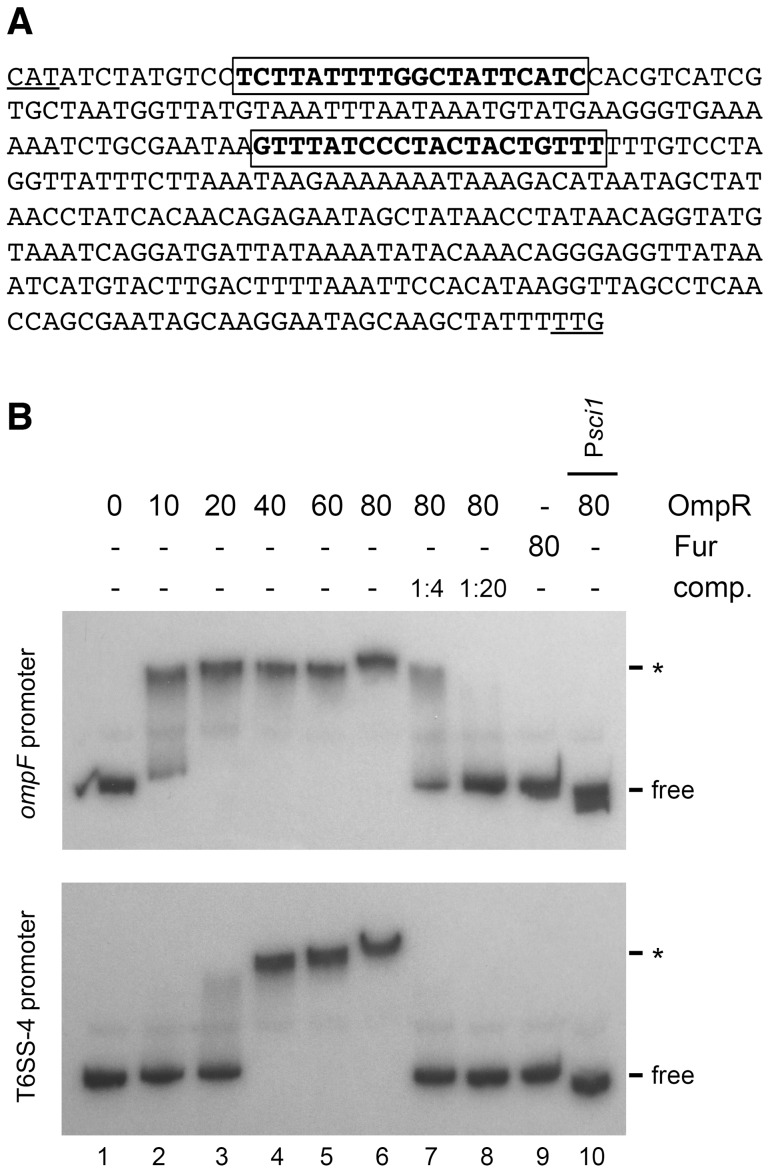
OmpR binds to the promoter region of T6SS-4. (A) Intergenic sequence upstream the first gene of the T6SS-4 operon. The TTG putative initiation codon is underlined, as the ATG initiation codon of the divergent gene upstream T6SS-4. The framed sequences in bold letters correspond to putative OmpR binding sites identified by *in silico* analyses using Virtual Footprint. A third OmpR binding site was experimentally identified upstream this intergenic region [63]. (B) Electrophoretic mobility shift assays of the *Y. pseudotuberculosis ompF* (upper panel) or T6SS-4 (lower panel) promoters using phosphorylated purified OmpR protein (lane 1, no protein; lane 2, 10 nM; lane 3, 20 nM; lane 4, 40 nM; lane 5, 60 nM; lane 6, 80 nM). Lanes 7 and 8: competition experiments with unlabelled T6SS-4 (upper panel) or *ompF* (lower panel) promoter PCR fragments at a promoter:competitor 1∶4 (lane 7) or 1∶20 (lane 8) ratio, in presence of 80 nM phosphorylated purified OmpR protein. Controls include incubation with the purified ferric uptake regulator Fur (lane 9, 80 nM) or incubation of the OmpR-independent enteroaggregative *E. coli sci-1* promoter PCR fragment (P*sci1*) with phosphorylated purified OmpR (lane 10, 80 nM). The positions of the free probes and of the shift fragments (*) are indicated.

*In silico* analysis of the T6SS-4 promoter region with Virtual Footprint suggests the existence of OmpR binding motifs (Figure 3A). To test whether the control by the OmpR protein was direct, we performed electrophoretic mobility shift assays. A recombinant N-terminally 6×His-TRX-tagged variant of the *Y. pseudotuberculosis* OmpR protein was purified to homogeneity by metal-affinity chromatography. The native OmpR*_Yps_* protein was obtained by tag proteolysis and gel filtration (see Material and Methods). OmpR being a transcriptional activator associated with a two-component system, its affinity for targets is dependent on its phosphorylation status [59]. Gel shift assays were therefore monitored in presence of acetyl-phosphate to promote OmpR auto-phosphorylation. The purified OmpR*_Yps_* protein bound to the promoter region of the *ompF* gene (Figure 3B, upper panel, lanes 1–6), a direct target of the OmpR response regulator [60]. This protein-DNA interaction was specific as no OmpR*_Yps_* binding was observed on the enteroaggregative *E. coli sci-1* promoter (Figure 3B, upper panel, lane 10) whose expression is regulated by the ferric uptake regulator [61] and independent on OmpR (the *sci1-lacZ* fusion shares comparable β-galactosidase activities in *E. coli* WT and Δ*ompR* strains; data not shown). A shift was observed when the T6SS-4 promoter was used (Figure 3B, lower panel, lanes 1–6). No shift was observed with the purified Fur protein in both *ompF* and T6SS-4 promoters (Figure 3B, lanes 9). The P*_T6SS-4_*-OmpR*_Yps_* shift was abolished when a competitor unlabelled DNA corresponding to the *ompF* promoter was used (Figure 3B, lower panel, lanes 7–8). Conversely, addition of the unlabelled T6SS-4 promoter decreased OmpR*_Yps_* binding on the *ompF* promoter (Figure 3B, upper panel, lanes 7–8). Taken together these results demonstrate that OmpR specifically binds to the T6SS-4 promoter region. However, it is worthy to note that the affinity for the purified OmpR protein is higher for the *ompF* promoter compared to the T6SS-4 promoter as (i) the *ompF* probe is retarded for lower OmpR concentrations (20 nM for *ompF* and 40 nM for T6SS-4) (compare upper and lower panels in Figures 3B, lanes 1–6) and (ii) the unlabelled *ompF* fragment has a stronger effect compared to the unlabelled T6SS-4 fragment in competition experiments (compare lanes 6–8).

### T6SS-4 Thermoregulation is Independent on *OmpR*

**Figure 4:**
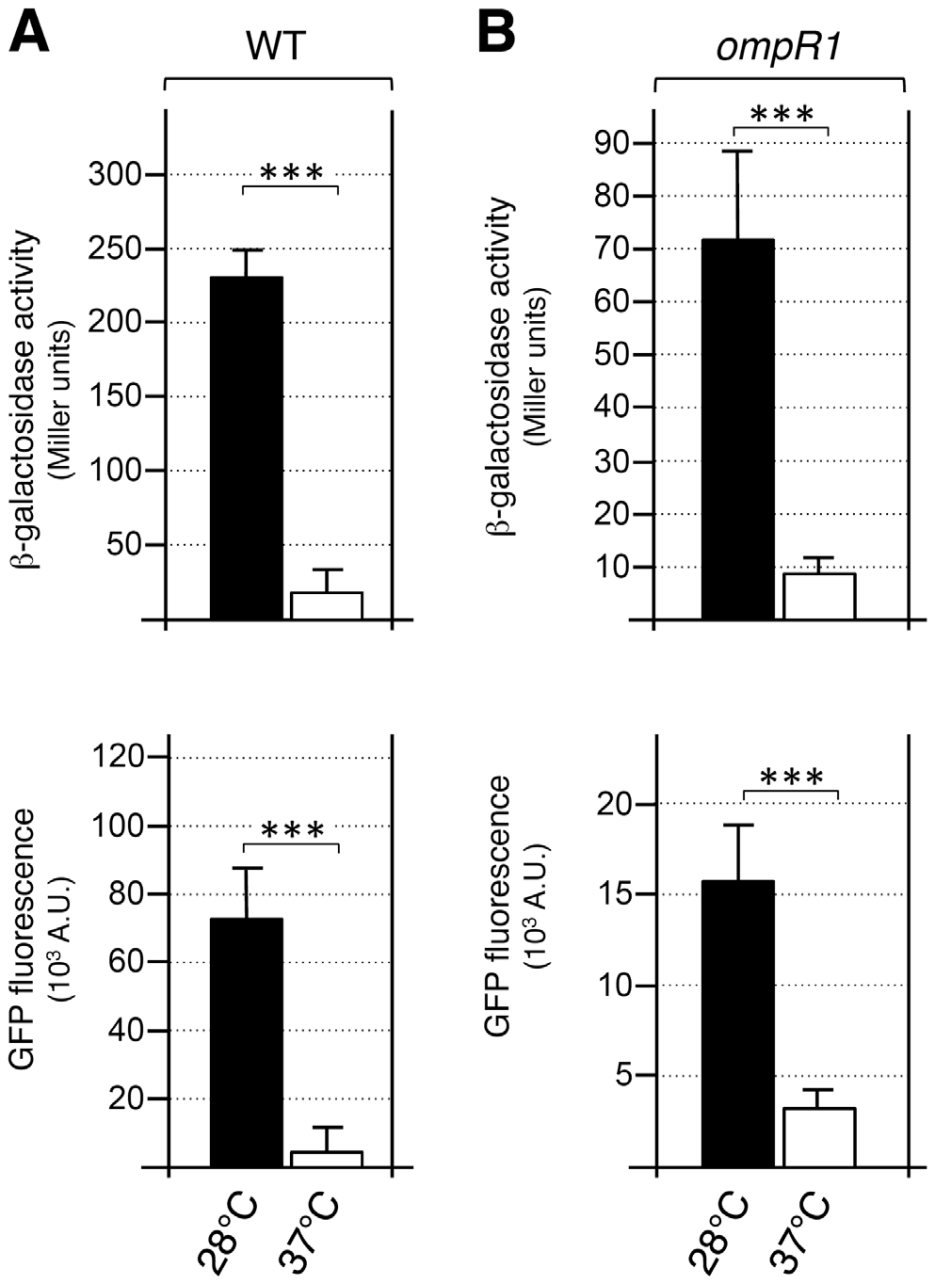
T6SS-4 thermoregulation is OmpR-independent. β-galactosidase activities (upper panel, in Miller units) and fluorescence levels (lower panel, in arbitrary units) of the T6SS-4 promoter fusions at 28°C and 37°C in the wild-type (A) and *ompR1* transposon (B) strains. Identical results were obtained for the *ompR2* transposon strain (data not shown). *p*-values obtained using paired Student’s *t-*test analyses are indicated (***, *p*≤0.0001).

Recently, Brzostek et al. reported that the temperature-dependent regulation of the *Y. enterocolitica* invasine *inv* gene was controlled by OmpR [62]. To test the role of OmpR in the T6SS-4 thermoregulation we compared the effect of the *ompR* mutations at 28°C and 37°C. Figure 4 shows that similar decreases in T6SS-4 gene cluster expression were observed in the WT (Figure 4A) and *ompR1* mutant (Figure 4B) strains at these two temperatures (8-fold decrease at 37°C compared to 28°C). These results show that OmpR has no role in the temperature-dependent expression of T6SS-4 and further suggest that T6SS-4 thermoregulation relies on additional regulatory mechanisms.

### T6SS-4 is Responsive to Cell Envelope and Osmotic Stresses in an OmpR-dependent Manner

**Figure 5:**
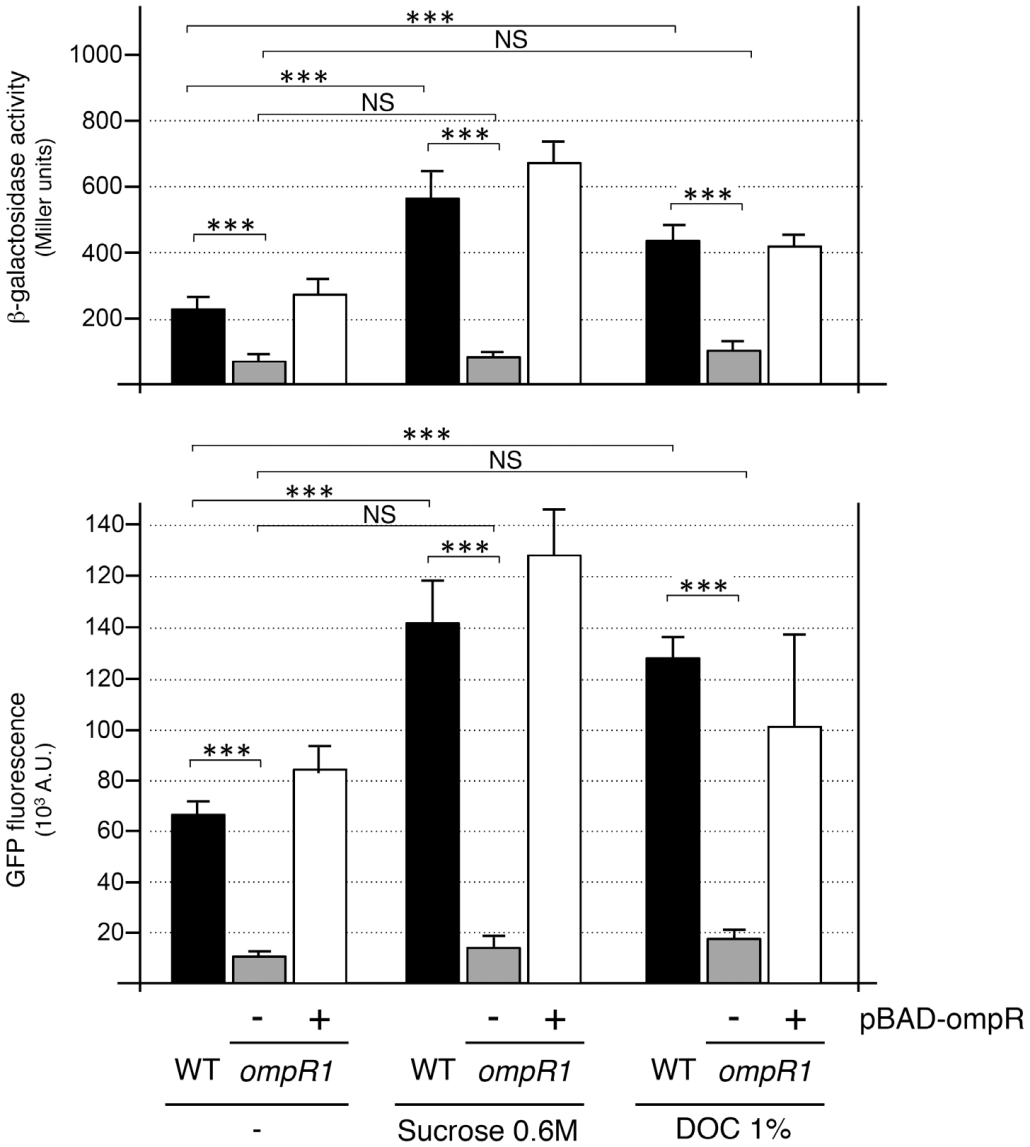
T6SS-4 gene expression is responsive to osmotic and cell envelope stresses through an OmpR-dependent mechanism. β-galactosidase activities (upper panel, in Miller units) and fluorescence levels (lower panel, in arbitrary units) of the T6SS-4 promoter in the WT or transposon *ompR1* strain carrying the pBAD24 empty vector (−) or pBAD-ompR (+) after exposure – or not – to 0.6 M Sucrose or to sodium deoxycholate (DOC) for 60 min. Identical results were obtained for the *ompR2* transposon strain (data not shown). *p*-values obtained using paired Student’s *t-*test analyses are indicated (NS [non significant], *p*>0.05; ***, *p*≤0.0001).

The EnvZ-OmpR two-component system regulates target genes in response to various stresses including cell envelope damages and high osmolarity [50], [52], [57]. Interestingly, LptD is an outer membrane protein required for LPS transport to the outer membrane [53], [55] suggesting that the *lptD* mutation induces a cell envelope stress. The observation that OmpR acts downstream LptD in the regulatory cascade suggests that the effect of LptD on T6SS-4 expression results from activation of OmpR engendered by a cell envelope stress. We therefore tested whether the expression of the T6SS-4 is modulated by cell envelope stresses engendered by exposure to high osmolarity or to sodium deoxycholate (DOC), a bile salt for 60 min. Figure 5 shows that the β-galactosidase activity and the GFP fluorescence levels of the wild-type strain increased 2.6- and 2-fold respectively in presence of 0.6 M sucrose (∼20%) and ∼2-fold in presence of 1% DOC. These increased activities of the promoter fusions in presence of osmotic or cell envelope stresses were dependent on OmpR as high osmolarity and bile salts had no effect on T6SS-4 expression in the *ompR1* transposon strain (identical results were obtained for the *ompR2* transposon strain).

### T6SS-4 is Required for Survival after Cell Exposure to an Osmotic Stress and for Resistance to Deoxycholate

The T6SS-4 being activated in high osmolarity conditions or in presence of bile salts we asked whether the *Y. pseudotuberculosis* T6SSs, and in particular T6SS-4, are required for survival after exposure to various stresses. Interestingly, several T6SSs have been shown implicated in the bacterial adaptation to environmental conditions: the *Vibrio anguillarum* T6SS regulates the stress response [22], the *V. cholerae* O1 T6SS is activated at high osmolarity [23] whereas the T6SS inhibits *Campylobacter* growth at high bile salt concentrations [24].

**Figure 6:**
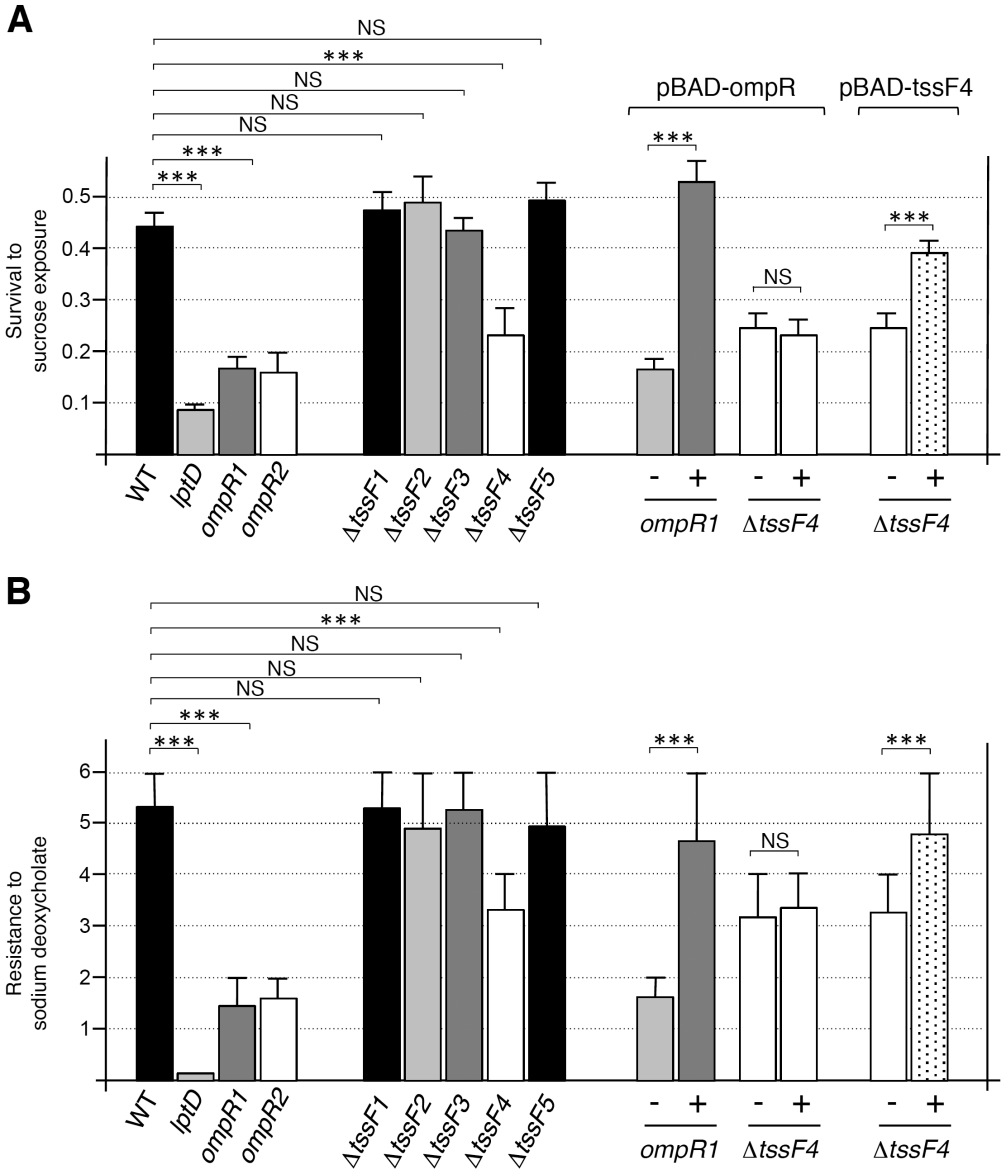
OmpR and T6SS-4 are required for survival after exposure to osmotic and cell envelope stresses. (A) Survival to exposure to osmotic stress. Viable bacteria (relative to the initial input) of the indicated strain counted after exposure for 60 min. to 0.6 M Sucrose. (B) Survival to cell envelope stress. Highest 10-fold serial dilution for which colonies are observable on LB plates supplemented with 1% sodium deoxycholate after 24 hours of incubation at 28°C. *p*-values obtained using paired Student’s *t-*test analyses are indicated (NS [non significant], *p*>0.05; ***, *p*≤0.0001).

To test survival upon osmotic stress exposure and resistance to a cell envelope stress, the wild-type *Y. pseudotuberculosis* strain and its derivatives in which the *tssF* gene, encoding an essential component of the secretion apparatus [25], [26], has been deleted (Δ*tssF1* [YpsIP31758_0316], Δ*tssF2* [YpsIP31758_2489], Δ*tssF3* [YpsIP31758_1361], Δ*tssF4* [YpsIP31758_3432] and Δ*tssF5* [YpsIP31758_0800]) were exposed to 20% of sucrose for one hour prior to counting viable cells or spotted onto LB agar plates supplemented with 1% DOC. Figure 6 shows that the survival to high osmolarity exposure (upper graph) and resistance to DOC (lower graph) were affected by the *tssF4*, *lptD* and *ompR* mutations while *tssF1*, *tssF2*, *tssF3* and *tssF5* cells displayed survival behaviours similar to the WT strain. It is worthy to note that the *lptD* and *ompR* mutant cells were more severely affected than *tssF4* cells. In trans-complementation experiments with pBAD24-OmpR, the survival of *ompR* and *lptD* mutant cells was restored while *tssF4* mutant cells remain sensitive to high osmolarity exposure and DOC. The *tssF4* phenotype was restored by production of a 6×His epitope-tagged TssF4 protein.

## Discussion

In many species T6SS gene clusters are found in several copies in the genome. Defining how these systems are regulated and if and how they cross-talk with each other is therefore important to better evaluate the contribution of the T6SS in the pathogenesis or fitness in different environments. Pathogenic *Yersinia* species usually contain 4 to 6 T6SS copies on the genome. In *Y. pseudotuberculosis* four complete and two incomplete T6SS can be identified [5], [47]. To gain insights into the regulatory mechanisms underlying expression of these systems, we have constructed reporter *lacZ* and *gfp* fusions to each of the putative promoter regions of the T6SS gene clusters and have used random mutagenesis to identify putative regulators. In this study, we report data regarding the control of T6SS-4 gene cluster expression. In agreement with previous studies [47]–[49], we showed that expression of the T6SS-4 gene cluster is enhanced at low temperature: a >10-fold increase of the reporter fusions is observed at 28°C compared to 37°C. Using random transposon mutagenesis we identified LptD and OmpR as T6SS-4 regulators. OmpR is the response regulator of the EnvZ-OmpR two-component system that responds to a variety of signals including osmotic and cell envelope stresses [50], [52]. Once phosphorylated by its cognate sensor kinase EnvZ, OmpR activates transcription of target genes. Indeed, T6SS-4 expression is induced in high osmolarity conditions or in presence of a bile salt, sodium deoxycholate, and *in vitro* gel shift assays demonstrated that purified phosphorylated OmpR binds to the T6SS-4 promoter. LptD (or Imp or OstA) is an outer membrane β-barrel protein involved in LPS transport and insertion into the outer leaflet of the outer membrane [55]. One hypothesis is that cell envelope defects conferred by the *lptD* mutation will activate the EnvZ-OmpR two-component system. This hypothesis is supported by the observation that overproduction of OmpR into *lptD* transposon mutant cells rescues T6SS-4 expression, suggesting that OmpR acts downstream LptD in the activation cascade. Our further experiments demonstrated that T6SS-4 is involved in resistance to high osmotic stresses and to bile salts.

While preparing this manuscript, a study reporting T6SS-4 regulation by OmpR was published by Zhang and collaborators [63]. While the authors of this study went in more details in several aspects, our results match with their data: (i) OmpR positively regulates T6SS-4 expression but does not affect expression of the other T6SS gene clusters and (ii) OmpR directly binds to the T6SS-4 promoter region. By using targeted mutagenesis, they conclusively show that the three OmpR binding sequences they identified in the T6SS-4 promoter region are all recognized as only mutations affecting the three boxes abolished OmpR binding and the OmpR-dependent regulation. However, they noted that the OmpR-mediated regulatory mechanism of the *Y. pseudotuberculosis* T6SS-4 gene cluster is probably more complex than expected. Among the three OmpR binding sites, two (O1 and O3) acts as activating operators while OmpR binding at O2 represses T6SS-4 transcription [63]. In this study, the authors further demonstrated that T6SS-4 is required for acid tolerance by regulating the intracellular pH in *Y. pseudotuberculosis* [63]. Taken together, the results from both studies demonstrate an OmpR-dependent regulation of the *Y. pseudotuberculosis* T6SS-4 gene cluster and point a role of this system for tolerance to different stresses (cell envelope, acid and osmotic). A role of the T6SS in stress resistance via sensing and activation of the stress response pathway has been demonstrated in *V. anguillarum* [22]. In *V. cholerae* O1, the activation of the T6SS has been shown to be dependent on the osmolarity of the milieu [23]. More recently, the *Campylobacter* T6SS has been shown to be involved to limit the growth when bile salts reach high concentrations [24]. As the role of a secretion system in stress resistance or sensing is not intuitive, further studies should be performed to understand the mechanistic bases for these phenotypes. Our survival experiments under exposure to osmotic stress showed that *ompR* transposon mutant cells were more severely affected than the *tssF4* cells. This suggests that OmpR activates additional genes involved in tolerance to high osmolarity. Osmoregulation of T6SS genes has been demonstrated in *V. cholerae*. The *vas* gene cluster of *V. cholerae* O1 is repressed at low osmolarity through the osmoregulatory repressor OscR [23]. Interestingly, the *V. cholerae* O1 *vas* gene cluster is activated at high osmolarity and low temperature [23]. As proposed by Ishikawa and coauthors, these results suggest that the *Y. pseudotuberculosis* T6SS-4 system might be activated in the environment rather than in the animal host. The observation that T6SS-4 is required for survival in high osmolarity conditions is probably beneficial to *Y. pseudotuberculosis* in the environment. Although the regulatory mechanisms of the *V. cholerae* O1 *vas* and *Y. pseudotuberculosis* T6SS-4 gene clusters appear similar, several differences can be noted. Notably, OscR represses the *vas* gene cluster at 37°C in *V. cholerae*. This is not the case in *Y. pseudotuberculosis*, in which OmpR has a positive effect on T6SS-4 expression at both 28°C and 37°C. Other regulators are probably involved in the control of T6SS-4 expression. The observation that OmpR has no role in the thermoregulation suggests that at least one additional regulator modulates T6SS-4 expression dependent on the temperature. T6SS-4 gene expression has also been proposed to be under the control of the RovA protein and of quorum sensing [47], [64]. One additional candidate is the histone-like H-NS protein. The T6SS-4 gene cluster has a deviating GC content (66% for the gene cluster, 69% for the promoter region compared to the 52% of the genome) that usually characterizes horizontally acquired DNA fragment which are preferred silenced targets of the H-NS protein. Interestingly, the OmpR protein has been shown to antagonize H-NS-mediated silencing of its own gene cluster in *Salmonella enterica* Typhimurium [65] and a number of studies have pointed regulatory cross-talks between H-NS and OmpR in Gram-negative bacteria [62], [66]. However, only three transposon mutant clones with lower expression were sequenced in this study and we can hypothesize that several other putative regulators might be identified by sequencing additional clones. Here, the transposon library was tested for decreased expression at 28°C. A screen to identify clones with higher expression at 37°C will also provide further insights into the control of T6SS-4 expression.

## Materials and Methods

### Bacterial Strains, Media and Chemicals

The *Y. pseudotuberculosis* IP31758 strain [67] was kindly provided by Pr. Elisabeth Carniel (Pasteur Institute, Paris, France). A spontaneous mutant of *Y. pseudotuberculosis* IP31758 resistant to nalidixic acid was obtained after growth in LB medium supplemented with Nal (5 µg/ml) for 16 hours at 28°C before spreading the liquid culture onto LB agar plates supplemented with Nal (20 µg/ml). This Nal^R^ strain (called RL21758-4) has been used throughout the study. *Escherichia coli* DH5α (New England Biolabs) was used for cloning procedures, S17-1 λpir or MFDpir [68] for mating experiments with *Y. pseudotuberculosis* and T7 Iq pLysS (New England Biolabs) for T7 promoter-driven protein production. Strains are listed in Table S1. Unless indicated, cells were grown in Luria broth (LB) at 28°C or 37°C. LB broth and MacConkey agar base were purchased from Difco. Plasmids were maintained by addition of ampicillin (100 µg/mL), chloramphenicol (40 µg/mL) or kanamycin (50 µg/mL). Gene expression was induced by 0.5 mM isopropyl-β-thio-galactoside (IPTG) or 0.1% arabinose. IPTG, arabinose, sucrose, DOC, X-Gal and ONPG were purchased from Sigma-Aldrich.

### Plasmid Construction

Plasmids and oligonucleotides are listed in Tables S1 and S2 respectively.

#### Suicide plasmids for *lacZ* fusions

Putative promoter regions were amplified from *Y. pseudotuberculosis* IP31758 chromosomal DNA with oligonucleotide pairs introducing restriction sites and PCR products were ligated into the pFUSE vector [69].

#### Suicide plasmids for *gfp* fusions

To construct chromosomal promoter-*gfp* fusions, a vector allowing insertion at the *ara* locus was designed as previously done for insertion of single-copy *lacZ* fusions in *Y. enterocolitica* [70]. Two 1.5-kb fragments (‘*araHG*’ and ‘*araBA*’) were amplified by PCR from *Y. pseudotuberculosis* IP31758 chromosomal DNA and ligated into the *Sac*I and *Spe*I sites of pSR47S [71] with a *Not*I site between them to yield pRL40. Next, promoterless *gfpmut2* was amplified from pUA66 [72] with oligonucleotides introducing *Not*I, *Nhe*I and *Bgl*II restriction sites upstream *gfpmut2*, and *Not*I downstream, and inserted into the *Not*I site of pRL40 to yield pRL44, in which the start codon of *gfpmut2* is immediately downstream the '*araGH*’ fragment. The putative promoter PCR fragments were then cloned into the *Nhe*I and *Bgl*II sites of pRL44. In these constructs, the promoters are fused to *gfpmut2* and can be inserted into the *Y. pseudotuberculosis* genome at the *ara* locus.

#### Suicide plasmids for construction of Δ*tssF* mutant strains

The *Y. pseudotuberculosis* Δ*tssF* in-frame deletion mutants were made using the *sacB* ^+^ allelic exchange plasmid pRE112 [73]. Two 0.5-kb fragments corresponding to immediately upstream and downstream regions of each *tssF* genes were PCR-amplified and fused by overlapping PCR, and cloned into the *Sac*I and *Kpn*I sites of pRE112.

For OmpR overproduction, the sequence encoding the *ompR* gene of *Y. pseudotuberculosis* was PCR amplified and cloned into pBAD24 [58] by RF cloning [74]. For TssF4 production, the sequence encoding the *tssF4* gene of *Y. pseudotuberculosis* fused to a 6×His coding sequence was PCR amplified and cloned between the *Kpn*I and *Pst*I sites of pBAD18-Kan [58]. In these constructs, the *ompR* and *tssF4* genes are under the control of the arabinose-inducible P*araBAD* promoter.

For OmpR protein purification, the sequence encoding the *ompR* gene of *Y. pseudotuberculosis* was PCR amplified and cloned into the Gateway™ destination pETG-20A vector (kindly provided by Dr. Arie Geerlof, European Molecular Biology Laboratory (EMBL), Hamburg, Germany) according to standard Gateway™ protocols. In this construct, OmpR is fused to an N-terminal hexahistidine-tagged thioredoxin (TRX) followed by a tobacco etch virus (TEV) protease cleavage site.

### Strains Construction

#### Insertion of *lacZ* fusions using the pFUSE plasmid derivatives

The pFUSE constructs were transferred to *Y. pseudotuberculosis* RL31758-4 by conjugation using S17-1 λpir as donor. Cultures of donor and recipient strains were cultivated in LB medium (at 37°C for donor strain, 28°C for recipient strain) with agitation until the end of the growth exponential phase. 1 ml of each culture were collected. Cells were harvested by centrifugation and then resuspended in 1 ml of 10 mM MgSO_4_. Mating was performed by mixing 400 µl of donor (Cm^R^) and 400 µl of recipient strains (Nal^R^) with 2.2 ml of 10 mM MgSO_4_. Cells were recovered by filtration on a 25-mm 0.45-µm pore size membrane. The filter was then placed on LB agar plates and incubated for 16 h at 28°C. Cells were collected and serial dilutions spread onto LB agar plates supplemented with Nal and Cm to select RL31758-4 transconjuguants. The pFUSE plasmid being unable to replicate into *Y. pseudotuberculosis* it integrated onto the chromosome by homologous recombination at the chromosomal locus. Proper integration of the plasmid was verified by colony PCR.

#### Insertion of *gfp* fusions using the pRL44 plasmid derivatives

The pRL44 derivatives were transferred to RL31758-4 by conjugation using S17-1 λpir as donor as described above. Proper integration of pRL44 into the chromosome disrupting the *ara* locus, plasmid integrants were identified by their Kan^R^ and *ara* ^−^ phenotypes on MacConkey-arabinose agar plates. Lost of the plasmid carrying the *sacB* counter-selectable marker was performed by streaking integrants on LB agar containing 10% sucrose. The Kan^s^ and *ara* ^−^ phenotypes of sucrose-resistant clones were verified on LB-Kan plates and MacConkey-arabinose agar plates respectively, and proper insertion of the fusion was verified by colony PCR.

#### Construction of Δ*tssF* mutant strains using the pRE112 plasmid derivatives

The pRE112 derivatives were transferred from S17-1 **λ**pir to *Y. pseudotuberculosis* RL31758-4 and plasmid integrants were selected on chloramphenicol LB agar plates. Double crossing-over was achieved by selection for sucrose-resistant segregants on LB agar plates supplemented with 10% sucrose. Proper in-frame deletion of the *tssF* genes was verified by colony PCR.

### Transposon Mutagenesis and Identification of the Insertion Site

For transposon mutagenesis, plasmid pAJD428 [75] was tranferred by conjugation from MFDpir to the *Y. pseudotuberculosis* strain carrying *lacZ* and *gfp* fusion to the T6SS-4 promoter (RL31758-41). MFDpir being devoid of the dihydrodipicolinate synthase *dapA* gene, it requires diaminopimelic acid (DAP) to the culture medium [68]. Recipient and donor strains were mixed in 10 mM MgSO_4_ as described above. Cells were immobilized on a 25-mm 0.45-µm pore size membrane filter disposed on LB agar plates supplemented with 0.3 mM DAP and incubated for 16 h at 28°C. Cells were collected and serial dilutions spread onto LB agar plates supplemented with Nal, Kan, IPTG and X-Gal (40 µg/ml). Colonies with decreased *lac* expression were selected and further tested for fluorescence levels. The conditional R6K replication origin of the transposon was used to obtain replicative plasmids. Briefly, candidate transposon mutant strains were grown overnight at 28°C and chromosomal DNA were purified using the Tissue and Blood DNA purification kit (Qiagen). 200 ng of chromosomal DNA were digested by EcoRI. After EcoRI heat-inactivation, fragments were ligated using the T4 DNA ligase (New England Biolabs) and the ligation mixture was electroporated into CC118λpir. Plasmids were purified from Kan^R^ clones and the transposon-chromosome junction was sequenced.

### β-galactosidase Activities and Fluorescence Levels

β-Galactosidase enzyme activity was measured in permeabilized cells harvested at an OD_600 nm_ of ∼2 as described previously using ortho-nitro-phenyl-β-D-galactopyrannoside (ONPG) as substrate [76]. Individual cultures were assayed in triplicate, and reported values (in Miller units) are averaged from three independent cultures.

#### Fluorescence levels

Cell cultures were diluted 10-fold into LB and 150 µl were transferred into wells of a black 96-well plate (Greiner). Absorbance at 600 nm and fluorescence (excitation: 485 nm; emission: 530 nm) were measured with a TECAN infinite M200 microplate reader. The relative fluorescence is expressed as the intensity of fluorescence divided by the absorbance at 600 nm, after subtracting the values of a blank sample. Individual cultures were assayed in triplicates and reported values are averaged from three independent cultures.

### Cell Survival to Osmotic Stress

Cells were grown at 28°C in LB with shaking to an OD_600 nm_∼0.6. 10^3^ cells were incubated for 60 min. with or without 0.6 M Sucrose (∼20%) at 28°C without shaking and then plated on LB plates. For induction from pBAD vectors, 0.1% of arabinose was added in the medium 60 min. prior to Sucrose addition. After overnight incubation at 28°C, colony forming units (cfu) were counted and cell survival was expressed as % of cfu after sucrose exposure relative to cfu without sucrose treatment.

### Cell Survival to Sodium Deoxycholate

Cells were grown at 28°C in LB with shaking to an OD_600 nm_∼0.6, harvested and resuspended in LB to an OD_600 nm_ of 0.5. 10 µl of 10^−1^ to 10^−6^ serial dilutions were spotted on LB agar plates supplemented with 1% DOC. For induction from pBAD vectors, 0.1% of arabinose was added in the medium 60 min. prior to cell resuspension, and 0.1% of arabinose was added in LB-DOC plates. Values are reported as the highest dilution for which colonies can be observed after 24 hours of growth at 28°C.

### Statistical Analyses

To determine whether two sets of data were significantly different, paired Student's *t*-tests were performed using the T.TEST function in Excel. *p*-values obtained from these statistical analyses are reported in the figures (*p*>0.05 indicated by NS [non significant], *p*≤0.01 indicated by *, *p*≤0.001 by **, and *p*≤0.0001 by ***).

### Purification of the *Y. pseudotuberculosis OmpR* Protein

pETG20-OmpR was transformed into the *E. coli* T7 Iq pLysS expression strain. Cells were grown at 37°C in terrific broth to an OD_600_ ∼ 0.9 and *ompR* expression was induced by addition of 0.5 mM IPTG for 16 hours at 17°C. Cells were harvested and resuspended in Tris-HCl 20 mM (pH8.0), NaCl 150 mM. Lysozyme was added (0.25 mg/ml) and cells were broken by sonication. The soluble proteins were separated from inclusion bodies and cell debris by centrifugation 30 min at 20,000×*g*. The TRX-6×His-TEV-OmpR fusion (42 kDa) was purified using Ni^2+^ affinity chromatography (HisTrap 5 ml GE Healthcare on an AKTA FPLC system) and eluted with a step gradient of imidazole. After proteolysis by a Hexahistidine-tagged TEV protease (1∶10 (w/w) protease:protein ratio) for 16 hours at 4°C, the TEV protease and contaminants were arrested on a second Ni^2+^ affinity column and the native OmpR protein (27 kDa) was collected in the flow through and separated on a preparative Superdex 200 gel filtration column (GE Healthcare) equilibrated in 20 mM Tris-HCl (pH 8.0), 150 mM NaCl. The final concentration of the OmpR solution was 1.5 mg/mL.

### Electrophoretic Mobility Shift Assays (EMSA)

Radiolabeled probes were generated by polymerase chain reaction (PCR) using a mix of dNTPs supplemented with [α-^32^P]dGTP (5 µCi per PCR in a total volume of 50 µl; Perkin-Elmer), and purified using the Wizard Gel and PCR clean-up kit (Promega). The EMSA protocol with the purified, phosphorylated OmpR protein was adapted from previously published protocols [77], [78]. To induce OmpR autophosphorylation, the protein was incubated for 30 minutes with 25 mM acetyl phosphate in Tris-HCl 10 mM (pH7.4), KCl 50 mM, Dithio threitol (DTT) 0.5 mM, MgCl_2_ 1 mM, Glycerol 4%. Radiolabeled PCR products (4 nM final concentration) were incubated in a final volume of 12 µL of Tris-HCl 10 mM (pH7.4), KCl 50 mM, DTT 0.5 mM, MgCl_2_ 1 mM, Glycerol 4%, BSA 50 µg/mL, sonicated salmon sperm DNA 50 µg/mL, acetyl phosphate 25 mM in presence of various concentrations of the phosphorylated OmpR protein. The mixtures were incubated for 20 minutes at 25°C and then loaded on a pre-run 8% non denaturing polyacrylamide (Tris-borate) gel. DNA and DNA-complexes were separated at 80 V in Tris-Borate buffer (45 mM Tris base, 45 mM boric acid, 100 µM MnCl_2_ buffer). Gels were fixed in 10% trichloro-acetic acid for 10 minutes, and exposed to Kodak BioMax MR films.

## Supporting Information

Table S1 **Strains and plasmids used in this study.**
(DOCX)

Table S2 **Oligonucleotides used in this study.**
(DOCX)

## References

[1] Bingle LE, Bailey CM, Pallen MJ (2008) Type VI secretion: a beginner's guide. Curr Opin Microbiol 11: 3–8. 18289922 10.1016/j.mib.2008.01.006

[2] Cascales E (2008) The Type VI secretion toolkit. EMBO Rep 9: 735–741. 18617888 10.1038/embor.2008.131 PMC2515208

[3] Filloux A, Hachani A, Bleves S (2008) The bacterial type VI secretion machine: yet another player for protein transport across membranes. Microbiology. 154: 1570–83. 10.1099/mic.0.2008/016840-0 18524912

[4] Records AR (2011) The Type VI secretion system: A multipurpose delivery system with a phage like machinery. Mol Plant Microbe Interact. 24: 751–7. 10.1094/MPMI-11-10-0262 21361789

[5] Boyer F, Fichant G, Berthod J, Vandenbrouck Y, Attree I (2009) Dissecting the bacterial type VI secretion system by a genome wide in silico analysis: what can be learned from available microbial genomic resources? BMC Genomics. 10: 104. 10.1186/1471-2164-10-104 PMC2660368 19284603

[6] Schwarz S, Hood RD, Mougous JD (2010) What is Type VI secretion doing in all those bugs? Trends Microbiol 18: 531–537. 20961764 10.1016/j.tim.2010.09.001 PMC2991376

[7] Pukatzki S, Ma AT, Sturtevant D, Krastins B, Sarracino D, et al (2006) Identification of a conserved bacterial protein secretion system in Vibrio cholerae using the Dictyostelium host model system. Proc Natl Acad Sci USA. 103: 1528–33. 10.1073/pnas.0510322103 PMC1345711 16432199

[8] Pukatzki S, Ma AT, Revel AT, Sturtevant D, Mekalanos JJ (2007) Type VI secretion system translocates a phage tail spike-like protein into target cells where it cross-links actin. Proc Natl Acad Sci USA 104: 15508–15513. 17873062 10.1073/pnas.0706532104 PMC2000545

[9] Ma AT, McAuley S, Pukatzki S, Mekalanos JJ (2009) Translocation of a Vibrio cholerae type VI secretion effector requires bacterial endocytosis by host cells. Cell Host Microbe. 5: 234–43. 10.1016/j.chom.2009.02.005 PMC3142922 19286133

[10] Suarez G, Sierra JC, Erova TE, Sha J, Horneman AJ, et al (2010) A type VI secretion system effector protein, VgrG1, from Aeromonas hydrophila that induces host cell toxicity by ADP ribosylation of actin. J Bacteriol. 192: 155–68. 10.1128/JB.01260-09 PMC2798274 19880608

[11] Durand E, Derrez E, Audoly G, Spinelli S, Ortiz-Lombardia M, et al (2012) Crystal Structure of the VgrG1 Actin Cross-linking Domain of the Vibrio cholerae Type VI Secretion System. J Biol Chem. 287: 38190–9. 10.1074/jbc.M112.390153 PMC3488088 22898822

[12] Hood RD, Singh P, Hsu F, Güvener T, Carl MA, et al (2010) A type VI secretion system of Pseudomonas aeruginosa targets a toxin to bacteria. Cell Host Microbe. 7: 25–37. 10.1016/j.chom.2009.12.007 PMC2831478 20114026

[13] Schwarz S, West TE, Boyer F, Chiang WC, Carl MA, et al (2010) Burkholderia type VI secretion systems have distinct roles in eukaryotic and bacterial cell interactions. PLoS Pathog 6: e1001068. 20865170 10.1371/journal.ppat.1001068 PMC2928800

[14] MacIntyre DL, Miyata ST, Kitaoka M, Pukatzki S (2010) The Vibrio cholerae type VI secretion system displays antimicrobial properties. Proc Natl Acad Sci U S A. 107: 19520–4. 10.1073/pnas.1012931107 PMC2984155 20974937

[15] Murdoch SL, Trunk K, English G, Fritsch MJ, Pourkarimi E, et al (2011) The opportunistic pathogen Serratia marcescens utilizes type VI secretion to target bacterial competitors. J Bacteriol. 193: 6057–69. 10.1128/JB.05671-11 PMC3194891 21890705

[16] Gueguen E, Cascales E (2013) Promoter Swapping Unveils the Role of the Citrobacter rodentium CTS1 Type VI Secretion System in Interbacterial Competition. Appl Environ Microbiol. 79: 32–8. 10.1128/AEM.02504-12 PMC3536073 23064344

[17] Brunet YR, Espinosa L, Harchouni S, Mignot T, Cascales E (2013) Imaging Type VI secretion mediated bacterial killing. Cell Rep. 3: 36–41. 10.1016/j.celrep.2012.11.027 23291094

[18] Russell AB, Hood RD, Bui NK, Leroux M, Vollmer W, et al (2011) Type VI secretion delivers bacteriolytic effectors to target cells. Nature. 475: 343–7. 10.1038/nature10244 PMC3146020 21776080

[19] Russell AB, Singh P, Brittnacher M, Bui NK, Hood RD, et al (2012) A widespread bacterial type VI secretion effector superfamily identified using a heuristic approach. Cell Host Microbe. 11: 538–49. 10.1016/j.chom.2012.04.007 PMC3358704 22607806

[20] English G, Trunk K, Rao VA, Srikannathasan V, Hunter WN, et al (2012) New secreted toxins and immunity proteins encoded within the Type VI secretion system gene cluster of Serratia marcescens.Mol Microbiol. 86: 921–36. 10.1111/mmi.12028 PMC3533786 22957938

[21] Aschtgen MS, Bernard CS, de Bentzmann S, Lloubes R, Cascales E (2008) SciN is an outer membrane lipoprotein required for type VI secretion in enteroaggregative Escherichia coli. J Bacteriol 190: 7523–7531. 18805985 10.1128/JB.00945-08 PMC2576670

[22] Weber B, Hasic M, Chen C, Wai SN, Milton DL (2009) Type VI secretion modulates quorum sensing and stress response in Vibrio anguillarum. Environ Microbiol. 11: 3018–28. 10.1111/j.1462-2920.2009.02005.x 19624706

[23] Ishikawa T, Sabharwal D, Bröms J, Milton DL, Sjöstedt A, et al (2012) Pathoadaptive conditional regulation of the type VI secretion system in Vibrio cholerae O1 strains. Infect Immun. 80: 575–84. 10.1128/IAI.05510-11 PMC3264300 22083711

[24] Lertpiriyapong K, Gamazon ER, Feng Y, Park DS, Pang J, et al (2012) Campylobacter jejuni type VI secretion system: roles in adaptation to deoxycholic acid, host cell adherence, invasion, and in vivo colonization. PLoS One. 7: e42842. 10.1371/journal.pone.0042842 PMC3428339 22952616

[25] Zheng J, Leung KY (2007) Dissection of a type VI secretion system in Edwardsiella tarda. Mol Microbiol 66: 1192–1206. 17986187 10.1111/j.1365-2958.2007.05993.x

[26] Zheng J, Ho B, Mekalanos JJ (2011) Genetic analysis of anti-amoebae and anti-bacterial activities of the type VI secretion system in Vibrio cholerae. PLoS One. 6: e23876. 10.1371/journal.pone.0023876 PMC3166118 21909372

[27] Mougous JD, Cuff ME, Raunser S, Shen A, Zhou M, et al (2006) A virulence locus of Pseudomonas aeruginosa encodes a protein secretion apparatus. Science 312: 1526–1530. 16763151 10.1126/science.1128393 PMC2800167

[28] Pell LG, Kanelis V, Donaldson LW, Howell PL, Davidson AR (2009) The phage lambda major tail protein structure reveals a common evolution for long-tailed phages and the type VI bacterial secretion system. Proc Natl Acad Sci USA 106: 4160–4165. 19251647 10.1073/pnas.0900044106 PMC2657425

[29] Leiman PG, Basler M, Ramagopal UA, Bonanno JB, Sauder JM, et al (2009) Type VI secretion apparatus and phage tail-associated protein complexes share a common evolutionary origin. Proc Natl Acad Sci USA 106: 4154–4159. 19251641 10.1073/pnas.0813360106 PMC2657435

[30] Bonemann G, Pietrosiuk A, Diemand A, Zentgraf H, Mogk A (2009) Remodelling of VipA/VipB tubules by ClpV-mediated threading is crucial for type VI protein secretion. EMBO J 28: 315–325. 19131969 10.1038/emboj.2008.269 PMC2646146

[31] Lossi NS, Dajani R, Freemont P, Filloux A (2011) Structure-function analysis of HsiF, a gp25-like component of the type VI secretion system, in Pseudomonas aeruginosa. Microbiology. 157: 3292–305. 10.1099/mic.0.051987-0 PMC3352280 21873404

[32] Cascales E, Cambillau C (2012) Structural biology of type VI secretion systems. Philos Trans R Soc Lond B Biol Sci. 367: 1102–11. 10.1098/rstb.2011.0209 PMC3297440 22411981

[33] Basler M, Pilhofer M, Henderson GP, Jensen GJ, Mekalanos JJ (2012) Type VI secretion requires a dynamic contractile phage tail-like structure. Nature. 483: 182–6. 10.1038/nature10846 PMC3527127 22367545

[34] Ma LS, Lin JS, Lai EM (2009) An IcmF family protein, ImpL_M_, is an integral inner membrane protein interacting with ImpK_L_, and its walker a motif is required for type VI secretion system-mediated Hcp secretion in Agrobacterium tumefaciens. J Bacteriol 191: 4316–4329. 19395482 10.1128/JB.00029-09 PMC2698499

[35] Aschtgen MS, Gavioli M, Dessen A, Lloubes R, Cascales E (2010) The SciZ protein anchors the enteroaggregative Escherichia coli Type VI secretion system to the cell wall. Mol Microbiol 75: 886–899. 20487285 10.1111/j.1365-2958.2009.07028.x

[36] Aschtgen MS, Thomas MS, Cascales E (2010) Anchoring the type VI secretion system to the peptidoglycan: TssL, TagL, TagP… what else? Virulence 1: 535–540. 21178498 10.4161/viru.1.6.13732

[37] Felisberto-Rodrigues C, Durand E, Aschtgen MS, Blangy S, Ortiz-Lombardia M, et al (2011) Towards a structural comprehension of bacterial type VI secretion systems: characterization of the TssJ-TssM complex of an Escherichia coli pathovar. PLoS Pathog. 7: e1002386. 10.1371/journal.ppat.1002386 PMC3213119 22102820

[38] Aschtgen MS, Zoued A, Lloubès R, Journet L, Cascales E (2012) The C-tail anchored TssL subunit, an essential protein of the enteroaggregative Escherichia coli Sci-1 Type VI secretion system, is inserted by YidC. Microbiologyopen. 1: 71–82. 10.1002/mbo3.9 PMC3426401 22950014

[39] Durand E, Zoued A, Spinelli S, Watson PJ, Aschtgen MS, et al (2012) Structural characterization and oligomerization of the TssL protein, a component shared by bacterial type VI and type IVb secretion systems. J Biol Chem. 287: 14157–68. 10.1074/jbc.M111.338731 PMC3340138 22371492

[40] Bernard CS, Brunet YR, Gueguen E, Cascales E (2010) Nooks and crannies in Type VI secretion regulation. J Bacteriol. 192: 3850–60. 10.1128/JB.00370-10 PMC2916374 20511495

[41] Leung KY, Siame BA, Snowball H, Mok YK (2011) Type VI secretion regulation: crosstalk and intracellular communication. Curr Opin Microbiol. 14: 9–15. 10.1016/j.mib.2010.09.017 20971679

[42] Silverman JM, Brunet YR, Cascales E, Mougous JD (2012) Structure and regulation of the type VI secretion system. Annu Rev Microbiol. 66: 453–72. 10.1146/annurev-micro-121809-151619 PMC3595004 22746332

[43] Mougous JD, Gifford CA, Ramsdell TL, Mekalanos JJ (2007) Threonine phosphorylation post-translationally regulates protein secretion in Pseudomonas aeruginosa. Nat Cell Biol. 9: 797–803. 10.1038/ncb1605 17558395

[44] Hsu F, Schwarz S, Mougous JD (2009) TagR promotes PpkA-catalysed type VI secretion activation in Pseudomonas aeruginosa. Mol Microbiol. 72: 1111–25. 10.1111/j.1365-2958.2009.06701.x PMC3402362 19400797

[45] Silverman JM, Austin LS, Hsu F, Hicks KG, Hood RD, et al (2011) Separate inputs modulate phosphorylation-dependent and -independent type VI secretion activation. Mol Microbiol. 82: 1277–90. 10.1111/j.1365-2958.2011.07889.x PMC3590308 22017253

[46] Casabona MG, Silverman JM, Sall KM, Boyer F, Couté Y, et al (2012) An ABC transporter and an outer membrane lipoprotein participate in posttranslational activation of type VI secretion in Pseudomonas aeruginosa. Environ Microbiol. 15: 471–86. 10.1111/j.1462-2920.2012.02816.x PMC3467343 22765374

[47] Zhang W, Xu S, Li J, Shen X, Wang Y, et al (2011) Modulation of a thermoregulated type VI secretion system by AHL-dependent quorum sensing in Yersinia pseudotuberculosis. Arch Microbiol. 193: 351–63. 10.1007/s00203-011-0680-2 21298257

[48] Robinson JB, Telepnev MV, Zudina IV, Bouyer D, Montenieri JA, et al (2009) Evaluation of a Yersinia pestis mutant impaired in a thermoregulated type VI-like secretion system in flea, macrophage and murine models. Microb Pathog. 47: 243–51. 10.1016/j.micpath.2009.08.005 PMC2767420 19716410

[49] Pieper R, Huang ST, Robinson JM, Clark DJ, Alami H, et al (2009) Temperature and growth phase influence the outer-membrane proteome and the expression of a type VI secretion system in Yersinia pestis. Microbiology. 155: 498–512. 10.1099/mic.0.022160-0 19202098

[50] Pratt LA, Hsing W, Gibson KE, Silhavy TJ (1996) From acids to osmZ: multiple factors influence synthesis of the OmpF and OmpC porins in Escherichia coli. Mol Microbiol. 20: 911–7. 10.1111/j.1365-2958.1996.tb02532.x 8809744

[51] Cai SJ, Inouye M (2002) EnvZ-OmpR interaction and osmoregulation in Escherichia coli. J Biol Chem. 277: 24155–61. 10.1074/jbc.M110715200 11973328

[52] Kenney LJ (2002) Structure/function relationships in OmpR and other winged-helix transcription factors. Curr Opin Microbiol. 5: 135–41. 10.1016/s1369-5274(02)00310-7 11934608

[53] Braun M, Silhavy TJ (2002) Imp/OstA is required for cell envelope biogenesis in Escherichia coli. Mol Microbiol. 45: 1289–302. 10.1046/j.1365-2958.2002.03091.x 12207697

[54] Wu T, McCandlish AC, Gronenberg LS, Chng SS, Silhavy TJ, et al (2006) Identification of a protein complex that assembles lipopolysaccharide in the outer membrane of Escherichia coli. Proc Natl Acad Sci U S A. 103: 11754–9. 10.1073/pnas.0604744103 PMC1544242 16861298

[55] Ruiz N, Kahne D, Silhavy TJ (2009) Transport of lipopolysaccharide across the cell envelope: the long road of discovery. Nat Rev Microbiol. 7: 677–83. 10.1038/nrmicro2184 PMC2790178 19633680

[56] Darwin AJ, Miller VL (1999) Identification of Yersinia enterocolitica genes affecting survival in an animal host using signature-tagged transposon mutagenesis. Mol Microbiol. 32: 51–62. 10.1046/j.1365-2958.1999.01324.x 10216859

[57] Mizuno T, Mizushima S (1990) Signal transduction and gene regulation through the phosphorylation of two regulatory components: the molecular basis for the osmotic regulation of the porin genes. Mol Microbiol. 4: 1077–82. 10.1111/j.1365-2958.1990.tb00681.x 1700256

[58] Guzman LM, Belin D, Carson MJ, Beckwith J (1995) Tight regulation, modulation, and high-level expression by vectors containing the arabinose PBAD promoter. J Bacteriol. 177: 4121–30. 10.1128/jb.177.14.4121-4130.1995 PMC177145 7608087

[59] Aiba H, Nakasai F, Mizushima S, Mizuno T (1989) Phosphorylation of a bacterial activator protein, OmpR, by a protein kinase, EnvZ, results in stimulation of its DNA-binding ability. J Biochem. 106: 5–7. 10.1093/oxfordjournals.jbchem.a122817 2674113

[60] Yoshida T, Qin L, Egger LA, Inouye M (2006) Transcription regulation of ompF and ompC by a single transcription factor, OmpR. J Biol Chem. 281: 17114–23. 10.1074/jbc.M602112200 16618701

[61] Brunet YR, Bernard CS, Gavioli M, Lloubès R, Cascales E (2011) An epigenetic switch involving overlapping fur and DNA methylation optimizes expression of a type VI secretion gene cluster. PLoS Genet. 7: e1002205. 10.1371/journal.pgen.1002205 PMC3145626 21829382

[62] Brzostek K, Brzóstkowska M, Bukowska I, Karwicka E, Raczkowska A (2007) OmpR negatively regulates expression of invasin in Yersinia enterocolitica. Microbiology. 153: 2416–25. 10.1099/mic.0.2006/003202-0 17660406

[63] Zhang W, Wang Y, Song Y, Wang T, Xu S, et al (2013) A type VI secretion system regulated by OmpR in Yersinia pseudotuberculosis functions to maintain intracellular pH homeostasis. Environ Microbiol. 15: 557–69. 10.1111/1462-2920.12005 23094603

[64] Cathelyn JS, Crosby SD, Lathem WW, Goldman WE, Miller VL (2006) RovA, a global regulator of Yersinia pestis, specifically required for bubonic plague. Proc Natl Acad Sci U S A. 103: 13514–9. 10.1073/pnas.0603456103 PMC1569194 16938880

[65] Bang IS, Audia JP, Park YK, Foster JW (2002) Autoinduction of the ompR response regulator by acid shock and control of the Salmonella enterica acid tolerance response. Mol Microbiol. 44: 1235–50. 10.1046/j.1365-2958.2002.02937.x 12068808

[66] Jubelin G, Vianney A, Beloin C, Ghigo JM, Lazzaroni JC, et al (2005) CpxR/OmpR interplay regulates curli gene expression in response to osmolarity in Escherichia coli. J Bacteriol. 187: 2038–49. 10.1128/JB.187.6.2038-2049.2005 PMC1064031 15743952

[67] Eppinger M, Rosovitz MJ, Fricke WF, Rasko DA, Kokorina G, et al (2007) The complete genome sequence of Yersinia pseudotuberculosis IP31758, the causative agent of Far East scarlet-like fever. PLoS Genet. 3: e142. 10.1371/journal.pgen.0030142 PMC1959361 17784789

[68] Ferrières L, Hémery G, Nham T, Guérout AM, Mazel D, et al (2010) Silent mischief: bacteriophage Mu insertions contaminate products of Escherichia coli random mutagenesis performed using suicidal transposon delivery plasmids mobilized by broad-host-range RP4 conjugative machinery. J Bacteriol. 192: 6418–27. 10.1128/JB.00621-10 PMC3008518 20935093

[69] Bäumler AJ, Tsolis RM, van der Velden AW, Stojiljkovic I, Anic S, et al (1996) Identification of a new iron regulated locus of Salmonella typhi. Gene. 183: 207–13. 10.1016/s0378-1119(96)00560-4 8996108

[70] Maxson ME, Darwin AJ (2005) Improved system for construction and analysis of single-copy beta-galactosidase operon fusions in Yersinia enterocolitica. Appl Environ Microbiol. 71: 5614–8. 10.1128/AEM.71.9.5614-5618.2005 PMC1214615 16151161

[71] Merriam JJ, Mathur R, Maxfield-Boumil R, Isberg RR (1997) Analysis of the Legionella pneumophila fliI gene: intracellular growth of a defined mutant defective for flagellum biosynthesis. Infect Immun. 65: 2497–501. 10.1128/iai.65.6.2497-2501.1997 PMC175352 9169800

[72] Zaslaver A, Bren A, Ronen M, Itzkovitz S, Kikoin I, et al (2006) A comprehensive library of fluorescent transcriptional reporters for Escherichia coli. Nat Methods 3: 623–8. 16862137 10.1038/nmeth895

[73] Edwards RA, Keller LH, Schifferli DM (1998) Improved allelic exchange vectors and their use to analyze 987P fimbria gene expression. Gene. 207: 149–57. 10.1016/s0378-1119(97)00619-7 9511756

[74] van den Ent F, Löwe J (2006) RF cloning: a restriction-free method for inserting target genes into plasmids. J Biochem Biophys Methods. 67: 67–74. 10.1016/j.jbbm.2005.12.008 16480772

[75] Maxson ME, Darwin AJ (2004) Identification of inducers of the Yersinia enterocolitica phage shock protein system and comparison to the regulation of the RpoE and Cpx extracytoplasmic stress responses. J Bacteriol. 186: 4199–208. 10.1128/JB.186.13.4199-4208.2004 PMC421588 15205422

[76] Miller JH (1972) Experiments in molecular genetics. Cold Spring Harbor Laboratory, Cold Spring Harbor, N.Y.

[77] Huang KJ, Lan CY, Igo MM (1997) Phosphorylation stimulates the cooperative DNA-binding properties of the transcription factor OmpR. Proc Natl Acad Sci U S A. 94: 2828–32. 10.1073/pnas.94.7.2828 PMC20281 9096305

[78] Gao H, Zhang Y, Han Y, Yang L, Liu X, et al (2011) Phenotypic and transcriptional analysis of the osmotic regulator OmpR in Yersinia pestis. BMC Microbiol. 11: 39. 10.1186/1471-2180-11-39 PMC3050692 21345178

