# Supplementary material for: Expression of a Yersinia pseudotuberculosis Type VI Secretion System Is Responsive to Envelope Stresses through the OmpR Transcriptional Activator
Source: PLoS One. 2013 Jun 19;8(6):e66615. doi: 10.1371/journal.pone.0066615 (PMC3686713; doi:10.1371/journal.pone.0066615)
Supplement: Table S1 — Strains and plasmids used in this study. (DOCX) [file pone.0066615.s001.docx]

**Table S1. Strains and plasmids used in this study.**

*Yersinia pseudotuberculosis* IP31758 derivatives

RL31758-4 Nal^R^ derivative of *Yersinia pseudotuberculosis* IP31758 This study

RL31758-7 RL31758-4 Φ(*P_T6SS-2_-lacZYA*) This study

RL31758-8 RL31758-4 Φ(*P_T6SS-5_-lacZYA*) This study

RL31758-9 RL31758-4 Φ(*P_T6SS-1_-lacZYA*) This study

RL31758-12 RL31758-4 Φ(P*_0696_-lacZYA*) This study

RL31758-19 RL31758-4 Φ(*P_T6SS-4_-lacZYA*) This study

RL31758-20 RL31758-4 Φ(*P_T6SS-3rev_-lacZYA*) This study

RL31758-21 RL31758-4 Φ(*P_T6SS-3fwd_-lacZYA*) This study

RL31758-36 RL31758-4 Φ(*P_T6SS-3rev_-lacZYA*) Δ*araGFB*::[Φ(*P_T6SS-3rev_*-*gfpmut2*)] This study

RL31758-37 RL31758-4 Φ(*P_T6SS-2_-lacZYA*) Δ*araGFB*::[Φ(*P_T6SS-2_*-*gfpmut2*)] This study

RL31758-38 RL31758-4 Φ(*P_T6SS-5_-lacZYA*) Δ*araGFB*::[Φ(*P_T6SS-5_*-*gfpmut2*)] This study

RL31758-39 RL31758-4 Φ(*P_T6SS-1_-lacZYA*) Δ*araGFB*::[Φ(*P_T6SS-1_*-*gfpmut2*)] This study

RL31758-40 RL31758-4 Φ(*P_0696_-lacZYA*) Δ*araGFB*::[Φ(*P_0696_*-*gfpmut2*)] This study

RL31758-41 RL31758-4 Φ(*P_T6SS-4_-lacZYA*) Δ*araGFB*::[Φ(*P_T6SS-4_*-*gfpmut2*)] This study

RL31758-42 RL31758-4 Φ(*P_T6SS-3fwd_-lacZYA*) Δ*araGFB*::[Φ(*P_T6SS-3fwd_*-*gfpmut2*)] This study

RL31758-10 RL31758-4 Δ*tssF1* This study

RL31758-14 RL31758-4 Δ*tssF3* This study

RL31758-16 RL31758-4 Δ*tssF5* This study

RL31758-17 RL31758-4 Δ*tssF2* This study

RL31758-18 RL31758-4 Δ*tssF4* This study

*Escherichia coli* strains

DH5α *fhuA2* Δ*(argF-lacZ)U169 phoA glnV44 Φ80* Δ*(lacZ)M15*

*gyrA96 recA1 relA1 endA1 thi-1 hsdR17* Laboratory collection

W3110 F^-^ lambda^-^ IN(*rrnD-rrnE*)1 *rph*-1 Laboratory collection

CC118λpir *Δ(ara-leu) araD ΔlacX74 galE galK phoA20 thi-1 rpsE*

*rpoB argE* (Am) *recA1* λpir lysogen Laboratory collection

S17-1λpir *recA thi pro hsd*(R- M+)RP4::2-Tc::Mu::Km Tn7 λ*pir* lysogen Laboratory collection

MFD λpir MG1655 RP4-2-Tc::[ΔMu1::*aac(3)IV*-Δ*aphA*-Δ*nic*35
ΔMu2::*zeo*] Δ*dapA*::(*erm*-*pir*) Δ*recA* [68]

T7 Iq F' *proA^+^B^+^ lacI^q^zzf::Tn10*(Tet^R^)*/ fhuA2 lacZ::T7 gene1 [lon] ompT gal sulA11 R(mcr-73::miniTn10--*Tet^S^*)2 [dcm] R(zgb-210::Tn10--*Tet^S^*) endA1 Δ(mcrC-mrr)114::IS10*  New England Biolabs

Plasmids

pFuse Suicide vector, *lacZYA*, *mob*^+^ (RP4), *ori*R6K, Cm^R^ [69]

pRL1 ~0.5-kb *P_T6SS-1_* XbaI-BglII PCR fragment in pFUSE This study

pRL2 ~1-kb *P_T6SS-5_* XbaI-BglII PCR fragment in pFUSE This study

pRL6 ~1-kb *P_T6SS-2_* BglII PCR fragment in pFUSE This study

pRL16 ~0.6-kb *P*_0696_ XbaI-BglII PCR fragment in pFUSE This study

pRL24 ~0.7-kb *P_T6SS-4_* XbaI-BglII PCR fragment in pFUSE This study

pRL29 ~0.8-kb *P_T6SS-3rev_* BglII PCR fragment in pFUSE This study

pRL30 ~0.8-kb *P_T6SS-3fwd_* BglII PCR fragment in pFUSE This study

pUA66 KmR, sc101 *ori*, promoterless version of the GFP reporter plasmid [72]

pSR47S KmR, *ori*R6K, *mob*+ (RP4), *sacB*+ [71]

pRL40 ~3-kb’*araHG*’-NotI-’*araBA*’ SacI/SpeI PCR fragment in pSR47S This study

pRL44 ~0.8-kb promoterless *mut2gfp* NotI PCR fragment in pRL40 This study

pRL48 BglII *P_T6SS-3rev_* fragment of pRL29 in BglII pRL44 This study

pRL49 BglII *P_T6SS-3fwd_* fragment of pRL33 in BglII pRL44 This study

pRL51 *P_T6SS-1_* XbaI-BglII fragment of pRL1 in NheI-BglII pRL44 This study

pRL52 *P_T6SS-5_* XbaI-BglII fragment of pRL2 in NheI-BglII pRL44 This study

pRL53 *P_T6SS-2_* BglII fragment of pRL6 in BglII-linearized pRL44 This study

pRL54 *P_T6SS-4_* XbaI-BglII fragment of pRL24 in NheI-BglII pRL44 This study

pRL55 *P*_0696_ XbaI-BglII fragment of pRL16 in NheI-BglII pRL44 This study

pRE112 CmR, *ori*R6K, *mob*+ (RP4), *sacB1*+ [73]

pRL10 ~1-kb SacI-KpnI Δ*tssF1* PCR fragment in pRE112 This study

pRL12 ~1-kb SacI-KpnI Δ*tssF3* PCR fragment in pRE112 This study

pRL21 ~1-kb SacI-KpnI Δ*tssF5* PCR fragment in pRE112 This study

pRL22 ~1-kb SacI-KpnI Δ*tssF2* PCR fragment in pRE112 This study

pRL23 ~1-kb SacI-KpnI Δ*tssF4* PCR fragment in pRE112 This study

pAJD428 Km^R^, *ori*R6K, pTn*Mod*-RKm’-*lacI^q^*-*ptac* [75]

pBAD24 Amp^R^, *ori* pBR322, *araC*, *araBAD* promoter [58]

pBAD-ompR *Y. pseudotuberculosis* IP31758 *ompR* gene cloned into pBAD24 This study

pBAD18-Kan Kan^R^, *ori* pBR322, *araC*, *araBAD* promoter [58]

pBAD-tssF4 *Y. pseudotuberculosis* IP31758 *tssF4* gene with a C-terminal 6×His

epitope tag cloned into pBAD18-Kan This study

pETG20A Gateway^TM^ vector, T7 promoter, thioredoxin (TRX)-6His Arie Geerlof

pETG20-OmpR *Y. pseudotuberculosis* IP31758 *ompR* gene cloned into pETG20A This study
