# Supplementary material for: Expression of a Yersinia pseudotuberculosis Type VI Secretion System Is Responsive to Envelope Stresses through the OmpR Transcriptional Activator
Source: PLoS One. 2013 Jun 19;8(6):e66615. doi: 10.1371/journal.pone.0066615 (PMC3686713; doi:10.1371/journal.pone.0066615)
Supplement: Table S2 — Oligonucleotides used in this study. (DOCX) [file pone.0066615.s002.docx]

**Table S2. Oligonucleotides used in this study.**

Construction of pFUSE derivatives. ^a^

Amplification of putative T6SS-1 promoter

5’-ATATATCTAGACATTTTTAATATCCTTGATAGTTTATTTGATTTTGATCAAAGGTTAGTC

5’-TATATAAGATCTCACACGTCCTTTTAATCTCTAATAAGGGGTGTCAGC

Amplification of putative T6SS-2 promoter

5’-TATATAAGATCTGGAATTTGGGGTCCAATGGTACGGAAGTG

5’-TATATAAGATCTCAACGTTCCTTATTAAGGGAAGGGAATTAAGGAAATCC

Amplification of putative T6SS-3 promoter

5’-TATATAAGATCTTTACTGCCCCGGTTATTATGTCAGCTTTCTTTCG

5’-TATATAAGATCTGTTTTTTATCCTGGTCTTTTTAACGGCGTTCATGTG

Amplification of putative T6SS-4 promoter

5’-ATATATCTAGAGTATTGTCATGCATAAGAAGGCGTTG

5’-ATATAAGATCTAAATAGCTTGCTATTCCTTGCTATTCGC

Amplification of putative T6SS-5 promoter

5’-TATATATCTAGACATCACTCCACGCTGTATTGCGCATCAC

5’-TATATAAGATCTCGATTTTCCTTATTAGGGGAAGGAAACTTAAGAAATCC

Amplification of putative vgrG (YpsIP31758_0696) promoter

5’-ATATATCTAGATTTATGCGTAAAGGGTATCAGTATGC

5’-ATATAAGATCTATCTGCTCCATCCTGGAAGTTAATGCG

Construction of pRL40 ^a^

Amplification of ’*araHG*’-NotI-’*araBA*’

5’-CGGGAGCTCCGCATGTTATCGCCAAAGCGC

5’-CGGACTAGTATGATATACGCCAGGCCACG

5’-AGAGGCGGCCGCCGATATCTTGATTGCCGATGACC

5’-ATATCGGCGGCCGCCTCTGCCCGAGAAATTGAGC

Construction of pRL44 ^a, b, c^

Amplification of promoterless *mut2gfp*

5’-GAAGCGGCCGCA*AAAAGGCCATCCGTCAGGATGGCCTTCT*GCCTGCAGGTCTGGACAT**TTATT**

**TGTACAATTCATCCATACCATGGGTAATCCC**

5’-GAAGCGGCCGC*ATAAAACGAAAGGCTCAGTCGAAAG*GCTAGCTAGATCTTTAAGAAGGAGATAT

ACAT**ATGAGTAAAGGAGAAGAACTTTTCACTGGAG**

Verification of the Δ*araGFB*::[Φ(*gfpmut2*)] genotype

5’-ATCCCCTTCAGTTACGGCG

5’-TCGATACCCACGGCTTTACC

5’-AAATTTGTGCCCATTAACATCACC

5’-GGTCCTTCTTGAGTTTGTAACAGC

Construction of pRE112 derivatives ^a^

Construction of pRL10

5’-CGGGAGCTCCCGACGATGGCCGCGCTTCTTAATTGGG

5’-TTACATCAGTGCATGCTTTTCGAACGACATTATGTTTCCTGG

5’-ATGTCGTTCGAAAAGCATGCACTGATGTAAGTGTGGTTGAGG

5’-CCGGGTACCGGACGACCTCGCTACCTAACCCCACC

Construction of pRL12

5’-CGGGAGCTCCACCCGTCATTAATTAACCATTAGG

5’-TTACAGGACTCCTCGAAATTTGTGATCCACTTACTGCCCCGG

5’-GTGGATCACAAATTTCGAGGAGTCCTGTAATGTTGCCCGATG

5’-CCGGGTACCCGGTGTCATTAAGTTCTTGAGG

Construction of pRL21

5’-CGGGAGCTCGAAAAAGAATATCAGAAAGGATACAGG

5’-GACGACTTAACCACAGGAGAACGTTTGGAATGGGAAG

5’-AAACGTTCTCCTGTGGTTAAGTCGTCCATAGGGTTCTCAG

5’-CCGGGTACCAGGTGTTATCTTTGCCGC

Construction of pRL22

5’-CGGGAGCTCTTTCCGGATGGTGGCTCTCACAGTCG

5’-GACGACTTAACCACAGGAGAACGTTTGGAATGGGAAG

5’-AAACGTTCTCCTGTGGTTAAGTCGTCCATAGGGTTCTCAG

5’-CCGGGTACCCCAGAAAGCGCGATGTTGGGG

Construction of pRL23

5’-CGGGAGCTCTTAGGAGAGCAGTCTTCCAAACTGG

5’-GTTGACAGGCACTTTCGTTGGGCATGACGG

5’-CCAACGAAAGTGGCCTGTCAACTATGGCTAGCC

5’-CCGGGTACCCCGTACCCGATCCACATGTG

Construction of pBAD-ompR ^d^

5’-*CATACCCGTTTTTTTGGGCTAGCAGGAGGA*ATGCAAGAGAATCACAAGATTCTGGTCGTTGATG

5’-*CTCTAGAGGATCCCCGGGTACCATGGTGA*TCATGCTTTACTGCCGTCCGGCACAAAGAC

Construction of pBAD-tssF4 ^a, e^

5’-TCGAGGTACCTAACGCGCAGAGATAAAACAGGG

5’-AGCTCTGCAGCTA*GTGATGGTGATGGTGATG*GCCATAGTTGACAGGCCCCCA

Construction of pETG20-OmpR

5’-GGGGACAAGTTTGTACAAAAAAGCAGGCTTAGAAAACCTGTACTTCCAGGGTCAAGA

GAATCACAAGATTCTGGTCG

5’-GGGGACCACTTTGTACAAGAAAGCTGGGTTTATTATAATACGGAAACCTCAATCAGTG

CTTTACTGCCGTCCGGCAC

Sequencing transposon-chromosome junctions

5’-CGGCCGCACTTGTGTATAAG

Gel Shift assays

Amplification of the putative *Y. pseudotuberculosis ompF* promoter

5’-CTGCTGCATTAGCTGCACCAGCG

5’-GAGCGTTTAATCTCATATGTCACTGGGG

Amplification of the putative *Y. pseudotuberculosis* T6SS-4 promoter

5’-GGAAATATGTCACTCATATTATTGTCCATCCG

5’-GGATTTCGCCTCAGGCATTCCCAC

^a^ Restriction sites underlined.

^b^ Antiterminator sequence italicized.

^c^ Sequence for *gfp-mut2* amplification in bold letters.

^d^ Sequence annealing on the target plasmid italicized.

^e^ Hexa-histidine-coding sequence italicized.
